# GSK3B Overexpression Alleviates Posttraumatic Osteoarthritis in Mice by Promoting DNMT1-Mediated Hypermethylation of NR4A3 Promoter

**DOI:** 10.1155/2022/4185489

**Published:** 2022-06-14

**Authors:** Zhou Lv, Deping Sun, Xin Li, Gang Wu

**Affiliations:** ^1^Orthopedic Resident Standardized Training, Qingdao Municipal Hospital (Group), Qingdao 266000, China; ^2^Department of Orthopedic Trauma, Yantai Affiliated Hospital of Binzhou Medical University, Yantai 264000, China; ^3^Department of Rheumatology, 1st Affiliated Hospital of Jin Zhou Medical University, Jinzhou, 121000, China; ^4^Department of General Surgery, 1st Affiliated Hospital of Jin Zhou Medical University, Jinzhou, 121000, China

## Abstract

**Background:**

Glycogen synthase kinase 3*β* (GSK3B) is reported to be a protective factor for the degradation of chondrocytes by extracellular mechanisms. Nuclear receptor subfamily 4 group A member 3 (NR4A3) is a proinflammatory factor in osteoarthritis. Their regulation mechanism in posttraumatic osteoarthritis (PTOA) is not fully understood.

**Methods:**

GSK3B expression in the cartilage tissue of PTOA patients was analyzed by western blotting. IL-1*β*-induced chondrocytes were transfected with pcDNA-GSK3B, and then, the cell viability, apoptosis, expression of the chondrocyte extracellular matrix degradation-related genes MMP13, aggrecan, and type II collagen, and secretion of inflammatory factors TNF-*α* and IL-6 were detected. Co-IP was used to analyze the interaction between GSK3B and DNMT1. Ch-IP and methylation-specific PCR assays were used for methylation. Also, cells were transfected with pcDNA-GSK3B or together with pcDNA-NR4A3, as well as transfected with si-NR4A3, and then, cell functions were tested. Then, the mice subjected to destabilization of medial meniscus (DMM) surgery were intra-articular injected with 100 *μ*L of the following adeno-related virus vectors (empty vector, Ad-GSK3B, scrambled shRNA, and sh-NR4A3), respectively, and the virus titer was 2 × 10^8^ TU/mL. Cartilage integrity was evaluated by safranin O/fast green staining, HE staining, and Osteoarthritis Research Society International (OARSI) score.

**Results:**

The expression of GSK3B protein was downregulated in PTOA patients. GSK3B overexpression alleviated IL-1*β*-induced chondrocyte apoptosis and extracellular matrix degradation, as well as cartilage mineralization in PTOA model mice. NR4A3 overexpression reversed the effect of GSK3B on IL-1*β*-induced chondrocyte functions. GSK3B could recruit DNMT1 to the NR4A3 promoter region to promote the methylation of NR4A3 and inhibit the expression of NR4A3 protein. Similarly, NR4A3 interference alleviated cartilage degradation under stimulating conditions by inhibiting the activation of the JAK2/STAT3 signaling pathway.

**Conclusion:**

GSK3B recruits DNMT1 to the NR4A3 promoter region and inhibits the activation of the NR4A3-mediated JAK2/STAT3 signaling pathway, thereby alleviating PTOA.

## 1. Introduction

Knee osteoarthritis (OA) can be classified as nontraumatic in patients having no history of knee trauma or posttraumatic in patients who sustained a traumatic knee injury and subsequently developed knee OA. There are indications that the distribution of structural changes between the medial and lateral compartments of the joint is different between nontraumatic knee OA and posttraumatic knee OA [1]. One of the hallmarks of posttraumatic osteoarthritis (PTOA) is the irreversible degradation of articular cartilage after trauma and/or abnormal joint load, leading to joint dysfunction, pain, and movement limitation. In PTOA, the crosstalk between the extracellular matrix (ECM) of chondrocytes is disrupted, resulting in an imbalance in their anabolic and catabolic activities [2]. The catabolism of chondrocytes is increased, but the anabolic activity is not offset, which will lead to the degradation of ECM components and ultimately damage the mechanical properties of the overall tissue. In turn, this will further destroy the mechanically regulated chondrocyte homeostasis, leading to a vicious circle of irreversible cartilage degeneration [3].

Studies reported so far indicate that glycogen synthase kinase 3*β* (GSK3B) activity is necessary for the differentiation of chondrocytes and osteoblasts and the development of endochondral bone [4]. Inhibition of GSK3B leads to the loss of cartilage marker expression and the reduction of chondrocyte proliferation [5]. A study showed that Smurf2 interacts with GSK-3*β* and induces its ubiquitination and subsequent proteasome degradation, activating the *β*-catenin signaling pathway, thereby aggravating osteoarthritis in mice [6]. DNA methylation is an epigenetic trait that plays an important role in transcriptional regulation during embryonic development and postpartum tissue growth. Studies showed that DNA methylation plays an important role in the pathogenesis of OA [7, 8]. DNA methylation is associated with gene silencing and takes place in CpG dinucleotides clustered in islands at the promoter region of the gene. Furthermore, DNA methylation analyses of genes associated with OA demonstrated a correlation between methylation levels and gene expression (e.g., MMP13, IL-1*β*, and TNF-*α* promoters exhibited demethylation, favoring their overexpression in osteoarthritic chondrocytes) [9]. A study collected 17 human cell lines and 27 human bladder cancer tissues and found that GSK3B phosphorylation was positively correlated with the expression of DNMT1 protein. GSK3B enhances the stability of DNMT1 and maintains DNA methylation and chromatin structure, which may contribute to the growth of cancer cells [10], while the mechanism of GSK3B and DNMT1 protein has not been clearly reported in PTOA.

Nuclear receptor subfamily 4 (NR4A) consists of NR4A1 (Nur77), NR4A2 (Nurr1), and NR4A3 (NOR1). These molecules are also known as orphan receptors and are believed to be important regulators involved in cellular function and inflammation reaction [11]. It is reported that NR4A3 is highly expressed in human osteoarthritis cartilage tissue. Overexpression of NR4A3 could promote the expression of cartilage matrix-degrading enzymes such as MMP-3, MMP-9, INOS, and COX-2 in chondrocytes induced by IL-1*β*. In addition, NR4A3 knockdown could alleviate the stimulation of IL-1*β* on chondrocytes. NR4A3 plays a proinflammatory role in the development of OA [12]. A study showed that in acute myeloid leukemia (AML) cell lines and primary AML cells, the CpG site in the intragenic region that contains exon 3 of the tumor suppressor gene NR4A3 instead of the promoter region is highly methylated. After the DNA methylation level of the CpG site in the gene is reduced, DNA methyltransferase inhibitors could restore the expression of NR4A3 [13], while the effect of NR4A3 methylation level on PTOA is unclear.

JAKs are tyrosine kinases associated with cytoplasmic receptors and are the first signaling pathways involved in cytokine stimulation [14]. So far, four members of the JAK family have been identified: JAK1, JAK2, JAK3, and Tyk2. JAK1, JAK2, and Tyk2 are ubiquitously expressed and can be associated with many cytokine receptor subunits. Phosphorylated JAKs recruit STAT containing SH2 domain and phosphorylate STAT. Subsequently, STATs form a homologous or heterodimeric complex and transfer to the nucleus to regulate the transcription of specific genes [15]. It is reported that NR4A3 overexpression could increase the activity of JAK2/STAT3 signaling in the heart of mice after myocardial infarction and alleviate the inflammatory response after myocardial infarction [16].

Our current research focuses on the functions of GSK3B and NR4A3 in PTOA and their underlying mechanisms. In addition, we studied the potential signaling pathways of these effects, such as JNK2/STAT3. Our research provides new strategies and directions for the treatment of PTOA.

## 2. Materials and Methods

### 2.1. Human Sample

The current study was performed with the approval of the Ethics Committee of the 1st Affiliated Hospital of Jin Zhou Medical University. This study was according to the Helsinki Declaration, and all patients signed the informed consent with the approval of the review committee. Then, a total of 18 cases of knee cartilage tissues from patients with posttraumatic amputation and 25 cases of cartilage tissues from patients with PTOA of the knee were collected from May 2017 to August 2020. Patients with rheumatoid arthritis and septic arthritis were excluded from this study. The enrolled 25 PTOA patients included 15 males and 10 females with an average age of 51.26 years, ranging from 43 to 67 years old. There were 18 cases of traumatic amputation, including 11 males and 7 females with an average age of 53.67 years, ranging from 47 to 68 years old. The cartilage tissues of 18 amputation patients were used as the normal control group, and the GSK3B protein expression in the cartilage tissues of the normal control group and the PTOA group was detected, respectively.

### 2.2. DMM-Induced PTOA Mouse Model

A total of 50 healthy male C57BL/6 mice, aged from 5 to 8 weeks old, were randomly classified into 5 experimental groups: sham, posttraumatic oleanolic acid (PTOA)+vector, PTOA+adeno-related virus GSK3B overexpression vector (Ad-GSK3B), PTOA+scramble, and PTOA+adeno-related virus NR4A3 shRNA (sh-NR4A3). Each group comprised 10 mice. Animals were raised in constant temperature at 25°C, with 12/12 light-dark cycle and supplied with food and water ad libitum. To generate a posttraumatic OA model, destabilization of medial meniscus (DMM) surgery was performed on mice. Briefly, mice were put in general anaesthesia by intraperitoneal injection of an anaesthetic cocktail (ketamine (100 mg/mL): xylazine (20 mg/mL): saline 1 : 0.5 : 8.5). The medial meniscus was destabilized under general anaesthesia. Animals were allowed to recover on a heating pad until they are fully recovered from the anaesthetics. In the sham group, rats were anesthetized only. Immediately after the surgery, the joint was injected with 100 *μ*L of the following adeno-related virus vectors (empty vector, Ad-GSK3B, scrambled shRNA, and sh-NR4A3), respectively, and the virus titer was 2 × 10^8^ TU/mL. Sample size estimation was performed based on the difference in Osteoarthritis Research Society International (OARSI) histopathological score between the groups. At 4 weeks after the surgery, the mice were euthanized by inhalation of carbon dioxide and the joints were harvested. All surface-soft tissues (skin, muscle, etc.) were removed to isolate cartilage tissues of joints. All animal studies were carried out with the approval of the 1st Affiliated Hospital of Jin Zhou Medical University Animal Care following the ethical code of animal use.

### 2.3. Histopathological Analysis

The samples were fixed and embedded following standard protocols. Firstly, samples were cut into 5 mm sections using microtome, and sections were stained with haematoxylin and eosin (H&E) and safranin O/fast green according to standard staining protocols for evaluation of histomorphology of the knee sections. Secondly, the severity of OA was graded by a modified OARSI score (cartilage) by two independent observers (one carried out blinded assessment). The agreement between the data obtained by observers was assessed by Cohen's kappa coefficient using online calculator QuickCalcs from GraphPad. The average scores from two independent observers were obtained for analysis. ImageJ was used to measure the thickness of hyaline cartilage (HC). Finally, all H&E and safranin O images were taken using a Nikon Eclipse 80i Microscope (Nikon, Japan).

### 2.4. Primary Culture of Chondrocytes and Treatment

Mouse articular chondrocytes were isolated from femoral condyles and tibial plateaus of 5-day-old male C57BL/6J mice. Briefly, mice were sacrificed and disinfected in 75% alcohol for 10 min. The femur head was exposed under aseptic conditions, and the total articular cartilage was further isolated, collected, and cut into 1-3 mm pieces. Then, the tissue was digested with 0.25% trypsin and 0.2% collagenase II for 30 min and 5 h, respectively, at 37°C. Cells were then filtered through a 70 *μ*m cell strainer and washed 3 times with sterile phosphate-buffered saline (PBS). Afterward, the collected chondrocytes were seeded into culturing dishes in DMEM at 37°C and 5% CO_2_. Culture medium was changed every 2-3 days. To induce degeneration, chondrocytes were treated with 5 ng/mL recombinant human IL-1*β* (Roche, Branchburg, NJ) for 24 h.

For transfection, pcDNA negative control (vector), pcDNA-GSK3B, pcDNA-NR4A3, siRNA-GSK3B (si-GSK3B), siRNA-NR4A3 (si-NR4A3), and siRNA negative control (scramble) were purchased from Sangon (Shanghai, China). When reaching 80% confluence, mouse chondrocytes were transfected with the above agents for 48 h by using Lipofectamine 3000 (Invitrogen, Carlsbad, CA, USA); after transfection, cells were harvested and subjected to total RNA and protein extraction.

### 2.5. Cell Viability Assay

Chondrocytes were inoculated into 96-well plates at a concentration of 2 × 10^4^ cells/mL and cultured at 37°C. CCK-8 reagent (Beyotime Institute of Biotechnology, Haimen, China) was added into each well for 30 min at 37°C, and absorbance values were read at 490 nm.

### 2.6. Enzyme-Linked Immunosorbent Assay (ELISA)

The interleukin-6 (IL-6) and tumor necrosis factor *α* (TNF-*α*) concentrations were detected exactly in line with the procedures presented within the IL-6 kit (R&D Systems, Minneapolis, MN, USA) and TNF-*α* kit (R&D Systems, Minneapolis, MN, USA). Finally, after supplementation of stopping solution, the absorbance (*A*) of each sample was determined at the wavelength of 450 nm by a microplate reader (Thermo Fisher Scientific, Waltham, MA, USA).

### 2.7. Reverse Transcription-Quantitative PCR (RT-qPCR)

The total RNA was isolated from chondrocytes and the mouse knee joint tissues with TRIzol™ Reagent (Invitrogen, Carlsbad, CA, USA). The PrimeScript RT Reagent Kit (Takara Biotechnology, Dalian, China) was used to synthesis cDNA. Real-time PCR was conducted by using SYBR Premix Ex Taq™ Kit (Applied Biosystems, Foster City, CA, USA). The reaction was run in ABI7500 real-time PCR system (Applied Biosystems, Carlsbad, CA). The PCR reaction system contained 12.5 *μ*L of Taq DNA polymerase, 1.0 *μ*L of RT primer, and 1.0 *μ*L of DNA sample, and double-distilled H_2_O was used to make up the vacant volume. The qPCR cycling conditions consisted of 95°C for 1 min, 35 cycle amplification for 20 s at 95°C, 60 s at 59°C, and 20 s at 72°C, and followed by 1 min at 72°C. The primers used in this study were synthesized by Sangon Biotech (Shanghai, China), and the primer sequences were as follows: nuclear receptor subfamily 4 group A member 3 (NR4A3) F: 5′-CCG AGC TTT AAC AGA TGC AA-3′; R: 5′-AGC TTC TGG ACA CGT CAA TG-3′; matrix metalloproteinase 13 (MMP13) F: 5′-GCT GGA CTC CCT GTT G-3′; R: 5′-TCG GAG CCT GTC AAC T-3′; collagen II F: 5′-GGG AAT GTC CTC TGC GAT GAC -3′; R: 5′-GAA GGG GAT CTC GGG GTT G-3′; aggrecan F: 5′-GAT GTT CCC TGC AAT TAC CAC CTC-3′; R: 5′-TGA TCT CAT ACC GGT CCT TCT TCT G-3′; *β*-actin F: 5′-TCT GTG TGG ATT GGT GGC T-3′; R: 5′-TCA TCG TAC TCC TGC TTG CT-3′. Relative amounts of mRNA for specific genes were calculated using 2^-*ΔΔ*Ct^ values, and each sample was run in duplicate for 6 times. The mean value of each set of duplicates normalized to that of mouse GAPDH was used to calculate relative gene expression.

### 2.8. Western Blotting

Whole protein was extracted from cell lysates or mouse knee joint tissues and quantified using a Bicinchoninic Acid (BCA) Protein Assay Kit (Thermo Scientific, Rockford, IL, USA). Then, an equal amount of protein (30 *μ*g/lane) was separated by 10% SDS-PAGE and electrotransferred onto a PVDF membrane (Roche, Basel, Switzerland). The membrane was incubated with 5% skim milk at room temperature for 2 h and then incubated with primary antibodies purchased from Abcam (Cambridge, UK), including rabbit monoclonal anti-GSK3B (1 : 1000, ab93926), anti-phosphorylated JAK2 (p-JAK2, 1 : 3000, ab32101), anti-total JAK2 (t-JAK2, 1 : 5000, ab108596), anti-p-STAT3 (1 : 1000, ab68153), anti-t-STAT3 (1 : 2000, ab76315), anti-DNMT1 (1 : 1000, ab188453), rabbit polyclonal anti-NR4A3 (1 : 1000, ab94507), anti-MMP13 (1 : 3000, ab39012), anti-collagen II (1 : 2000, ab34712), anti-*β*-actin (1 : 2000, ab8227), and mouse monoclonal anti-aggrecan (1 : 2500, ab3773) antibodies overnight at 4°C. Then, the membrane was incubated with goat anti-rabbit IgG (1 : 3000, Abcam, ab6721) conjugated with horseradish peroxidase secondary antibody (HRP) for 2 h, and GAPDH was used as a loading control. Protein bands were visualized with an ECL chemiluminescence kit (BioTeke, Beijing, China) and quantified with ImageJ software (National Institutes of Health, Bethesda, Massachusetts, USA) and E-Gel imager (Thermo Fisher Scientific).

### 2.9. Methylation-Specific PCR Assay

The PCR was carried out with HotStar Taq polymerase (Qiagen, Dusseldorf, Germany). The conditions were as follows: initial incubation at 95°C for 4 minutes; 35 cycles of 95°C for 30 s, 70°C for 30 s, and 72°C for 30 s; PCR products were electrophoresed in 3% agarose gels and visualized by ultraviolet illumination. The sequence of NR4A3 MSP primer was constructed by Sangon (Shanghai, China) and presented as follows: methylated primer: 5′-TTC GGT TAA AAA TAG TTA GGT TCG A-3′ (forward) and 5′-ACG AAA TAA ATT CCC CTC GAT-3′ (reverse); unmethylated primer: 5′-TTT GGT TAA AAA TAG TTA GGT TTG A-3′ (forward) and 5′-AAT AAC AAA ATA AAT TCC CCT CAA T-3′ (reverse).

### 2.10. Flow Cytometry

A flow cytometry assay was performed using the annexin V-propidium iodide (PI) apoptosis kit according to the manufacturer's instructions (Invitrogen, Carlsbad, CA, USA). Chondrocytes were seeded in 6-well plates (1 × 10^6^ cells/well) and cultured for 48 h. Then, cells were harvested by trypsinization, washed with cold phosphate-buffered saline (PBS), and resuspended in binding buffer. Annexin V-fluorescein isothiocyanate (FITC) and PI were added to the suspension. The suspension was incubated for 15 min in the dark at room temperature, and then, the binding buffer was added. Finally, the samples were analyzed by flow cytometry.

### 2.11. Coimmunoprecipitation (Co-IP) Assay

Pierce™ Classic Bead Co-IP Kit (Thermo Fisher Scientific, Waltham, MA, USA) was used for Co-IP assay according to the manufacturer's instructions. After transfection, the 293T cells were washed with cold PBS, added 1 mL of PBS, and scraped the cells to a 1.5 mL EP tube, and draw 100 *μ*L from it as input. Then, the lysis buffer containing complete protease inhibitor was added to the remaining cell suspension, centrifuged at 14,000 × *g* at 4°C for 20 min. The supernatant was incubated with normal IgG (1 : 50, ab172730, Abcam) or anti-GSK3B (1 : 30, ab280376, Abcam) antibody. Then, the immune complex was combined with protein A-sepharose and analyzed by western blot.

### 2.12. Chromatin Immunoprecipitation Assay

Chromatin immunoprecipitation (Ch-IP) detection was performed by using an Enzymatic Chromatin IP kit (Invitrogen, Carlsbad, CA, USA) according to the manufacturer's instructions. Briefly, the chondrocyte lysate was crosslinked with 1% formaldehyde, and then, a protease inhibitor chip lysis buffer was added. Ultrasound was used to disrupt the lysate to obtain chromatin with an average size of 200-500 bp. Also, mouse knee joint tissues were crosslinked with 1% formaldehyde followed by adding the Ch-IP lysis buffer with protease inhibitors. Sonicate the tissue to obtain chromatin with an average size of 200-500 bp. And then after immunoprecipitation was incubation with anti-DNMT1 antibody and normal rat IgG overnight at 4°C. Immune complexes were collected with Protein G Agarose Beads (Cell Signaling Technology, Boston, MA, USA) after preincubation with salmon sperm DNA and BSA for 1 h at 4°C. The beads were washed and eluted with elution buffer. The elution was incubated at 65°C for 2 h to reverse the crosslinking after adjusting the NaCl concentration. The DNA was purified with a Thermo Scientific GeneJET Viral DNA kit (Thermo Fisher Scientific), amplified with the Platinum™ Taq DNA Polymerase High Fidelity and quantified with Applied Biosystems 7500 Real-Time PCR System.

### 2.13. Statistical Analysis

All data were expressed as mean ± standard deviation (SD) and analyzed with SPSS Statistics (IBM, Armonk, New York, USA). Comparisons between two groups were carried out using the Student's *t*-test. Differences between the groups were compared by one-way analysis of variance (ANOVA), and Duncan's method was used as a post hoc test. *P* < 0.05 was considered as statistically significant.

## 3. Results

### 3.1. GSK3B Is Downregulated in Articular Cartilage of Patients with Posttraumatic Osteoarthritis

**Figure 1:**
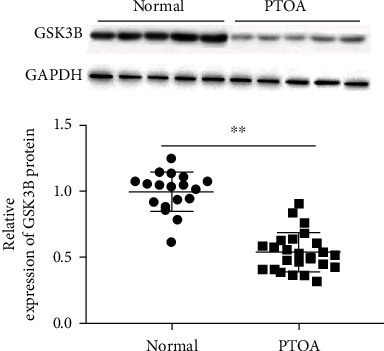
GSK3B protein is downregulated in articular cartilage of patients with posttraumatic osteoarthritis. We collected 18 cartilage tissues from amputation patients as a normal control group and 25 cartilage tissues from PTOA patients. Then, western blotting was used to detect the expression of GSK3B protein. Data is presented as the means ± SD for six independent experiments and analyzed by Student's *t*-test or ANOVA test. ^∗∗^*P* < 0.01.

A recent study showed that the inactivation of glycogen synthase kinase 3*β* (GSK3B) in human primary chondrocytes could lead to a decrease in cell elasticity and viscosity, as well as the destruction of cell microtubule networks [17]. We collected 18 cartilage tissues from amputation patients as a normal control group and 25 cartilage tissues from PTOA patients. Western blotting was used to detect the GSK3B protein expression in cartilage tissues of the normal control group and the PTOA group. The results showed that GSK3B was significantly downregulated in cartilage tissues of PTOA patients (Figure 1).

### 3.2. GSK3B Overexpression Alleviates IL-1*β*-Induced Degeneration of Mouse Chondrocytes

**Figure 2:**
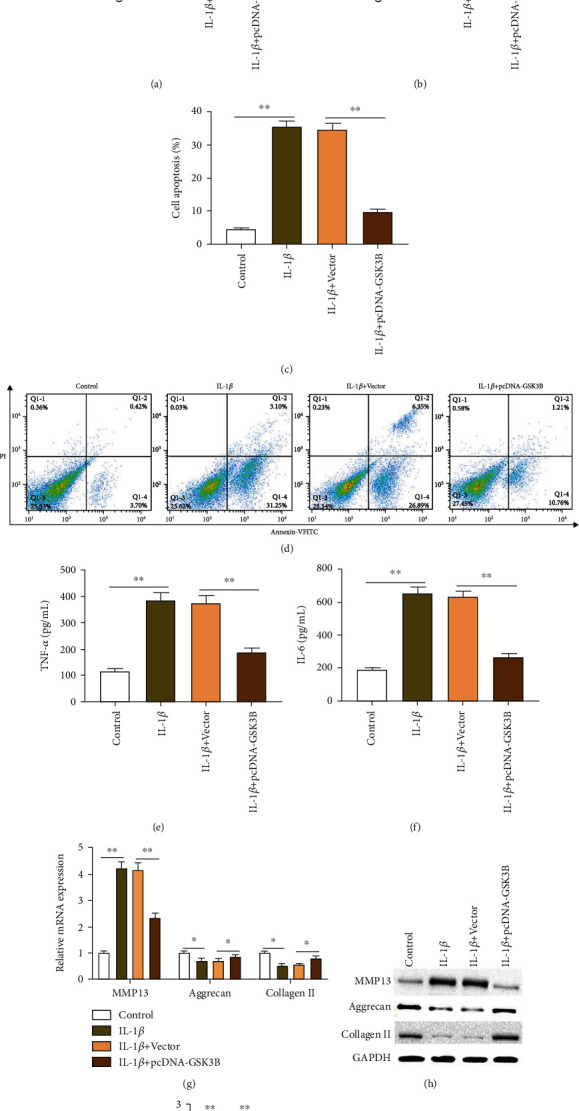
GSK3B overexpression alleviates IL-1*β*-induced degeneration of mouse chondrocytes. IL-1*β*-induced chondrocytes were transfected with 40 *μ*g/mL pcDNA-GSK3B for 48 h, and then, western blotting was used to detect the expression of GSK3B (a), MMP13, aggrecan, and type II collagen protein (h, i), CCK-8 assay was used to detect cell viability (b), flow cytometry was used to detect apoptosis (c, d), ELISA kits were used to detect the secretion of inflammatory factors TNF-*α* (e) and IL-6 (f), and RT-qPCR was used to detect the expression of MMP13, aggrecan, and type II collagen mRNA (g). Data is presented as the means ± SD for six independent experiments and analyzed by Student's *t*-test or ANOVA test. ^∗^*P* < 0.05 and ^∗∗^*P* < 0.01.

Next, IL-1*β*-induced chondrocytes were transfected with pcDNA-GSK3B, and then, cell viability, apoptosis, expression of aggrecan and collagen II, which are composed of extracellular matrix of chondrocytes, expression of matrix metalloproteinase 13 (MMP13), which is a marker for the degradation of extracellular matrix of chondrocytes, and the secretion of inflammatory factors IL-6 and TNF-*α* were detected. The results showed that compared with the control group, IL-1*β* treatment significantly reduced the cell viability (Figure 2(b)), the expression of GSK3B protein (Figure 2(a)), and the expression of aggrecan and collagen II mRNA and proteins (Figures 2(g)–2(i)) and increased cell apoptosis (Figures 2(c) and 2(d)), the secretion of inflammatory factors IL-6 and TNF-*α* (Figures 2(e) and 2(f)), and the expression of MMP13 mRNA and protein (Figures 2(g)–2(i)), while pcDNA-GSK3B transfection rescued the IL-1*β*-induced chondrocyte degradation.

### 3.3. GSK3B Promotes NR4A3 Promoter Methylation by Recruiting DNMT1

**Figure 3:**
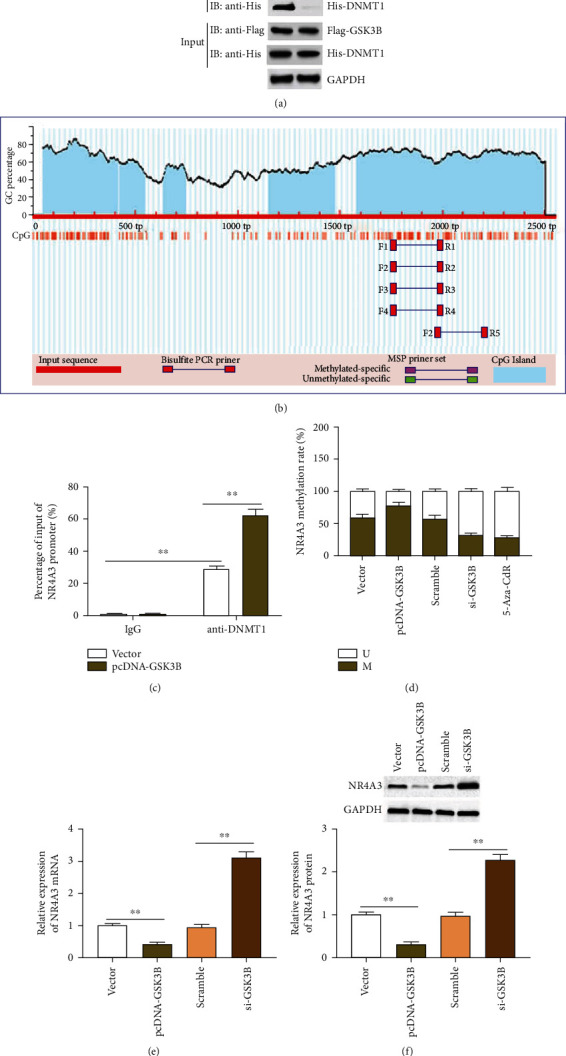
GSK3B promotes NR4A3 promoter methylation by recruiting DNMT1. Online prediction database HitPredict (http://www.hitpredict.org/index.html) was used to predict the binding of GSK3B and DNMT1. (a) Co-IP assay in 293T cells was used to show the binding between GSK3B and DNMT1. Whole-cell lysates were immunoprecipitated with anti-Flag, anti-His, or control anti-IgG antibody and analyzed by western blotting. (b) Online database MethPrimer was used to predict the CpG islands in the promoter region of NR4A3. (c) The enrichment of DNMT1 in the NR4A3 promoter region was detected by Ch-IP assay. (d) MSP-PCR assay was used to detect the methylation level of NR4A3 promoter region in chondrocytes. (e) RT-qPCR was used to detect the mRNA level of NR4A3. (f) Western blotting was used to detect the expression of NR4A3 protein. 5-aza-2′-deoxycytidine (5-Aza-CdR): DNA methylase inhibitor. Data is presented as the means ± SD for six independent experiments and analyzed by Student's *t*-test or ANOVA test. ^∗∗^*P* < 0.01.

We used the online database HitPredict to predict the proteins that interact with GSK3B and found that DNMT1 is one of them (Figure 3(a)). To elucidate the mechanisms of the GSK3B requirement, we tested whether DNMT1 and GSK3B interact with each other. The interaction between these two proteins was first detected by coimmunoprecipitation (Co-IP) in 293T cells using anti-Flag to pull down the Flag-tagged GSK3B and their interacting partner, which was detected by both antibodies in western blots, and the results indicated that Flag-GSK3B coimmunoprecipitated with His-DNMT1 (Figure 3(b)).

It is reported that NR4A3 is highly expressed in osteoarthritis, which may be related to the decrease of methylation level. We used the online database MethPrimer to predict whether there are CpG islands in the promoter region of NR4A3, and we found that there are multiple CpG islands in the promoter region of NR4A3, indicating that NR4A3 has a high possibility of being methylated (Figure 3(c)). To investigate the role of GSK3B in the methylation process of NR4A3, we conducted Ch-IP analysis and showed that DNMT1 bound to the promoter ofNR4A3, and GSK3B overexpression prominently promoted DNMT1 enrichment (Figure 3(d)). The MSP-PCR assay showed that GSK3B overexpression significantly promoted the methylation level of the NR4A3 promoter, while GSK3B interference resulted in a decrease in the methylation level of the NR4A3 promoter, which had the same effect as the methylation inhibitor 5-Aza-cdR (Figure 3(e)). Furthermore, compared with the vector group, GSK3B overexpression decreased the expression of NR4A3 mRNA and protein, and compared with the scramble group, GSK3B interference increased the expression of NR4A3 mRNA and protein (Figure 3(f)). These data suggested that GSK3B advances the methylation level of NR4A3 in chondrocytes by recruiting DNMT1.

### 3.4. Hypermethylation of NR4A3 Promoter Alleviates IL-1*β*-Induced Degeneration of Mouse Chondrocytes

**Figure 4:**
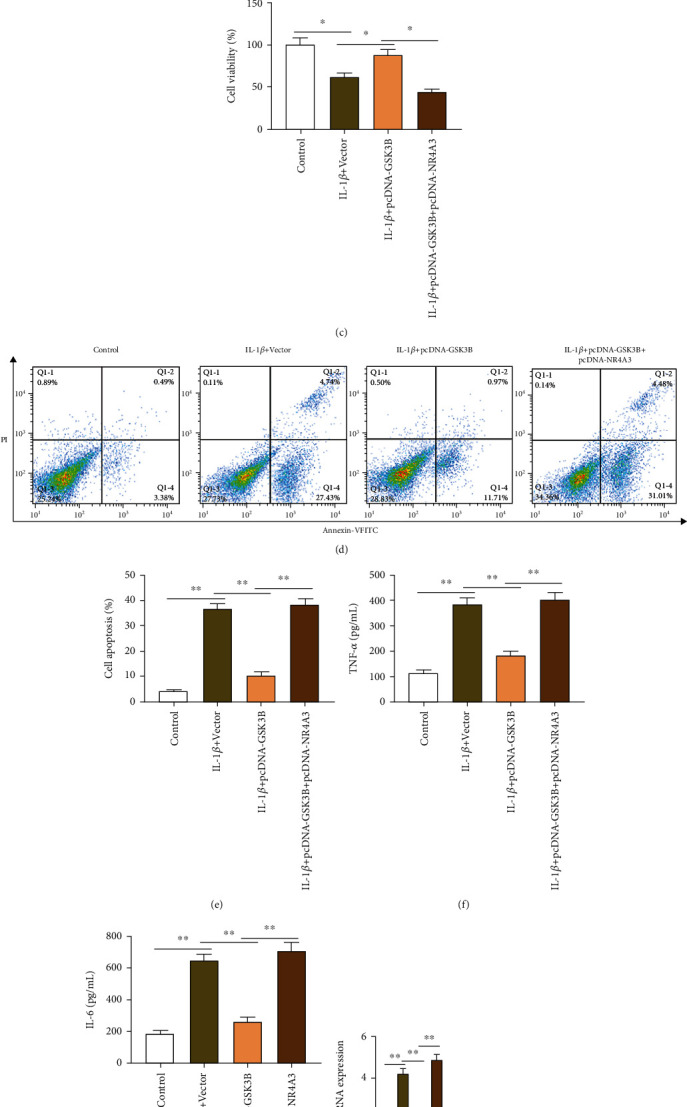
NR4A3 promoter hypermethylation alleviates IL-1*β*-induced degeneration of mouse chondrocytes. IL-1*β*-induced chondrocytes were transfected with pcDNA-GSK3B or together with pcDNA-NR4A3. (a) MSP-PCR assay was used to detect the methylation level of NR4A3 promoter region in chondrocytes. (b, h–j) Western blotting was used to detect the expression of NR4A3, MMP13, aggrecan, and type II collagen protein. (c) CCK-8 assay was used to detect cell viability. (d, e) Flow cytometry was used to detect apoptosis. (f, g) ELISA kits were used to detect the secretion of inflammatory factors TNF-*α* and IL-6. (h) RT-qPCR was used to detect the expression of MMP13, aggrecan, and type II collagen mRNA. Data is presented as the means ± SD for six independent experiments and analyzed by Student's *t*-test or ANOVA test. ^∗^*P* < 0.05 and ^∗∗^*P* < 0.01.

To investigate the effect of NR4A3 promoter methylation on IL-1*β*-induced chondrocyte degradation, IL-1*β*-induced chondrocytes were transfected with pcDNA-GSK3B or together with pcDNA-NR4A3, and then, the chondrocyte functions were tested. The MSP-PCR and western blotting experiments showed that compared with the control group, IL-1*β* stimulation reduced the methylation level of NR4A3 promoter in chondrocytes (Figure 4(a)) and increased the expression of NR4A3 protein (Figure 4(b)). Moreover, pcDNA-GSK3B transfection increased the methylation level of the NR4A3 promoter (Figure 4(a)), cell viability (Figure 4(c)), and the expression of aggrecan and collagen II mRNA and proteins (Figures 4(h)–4(j)) and reduced cell apoptosis (Figures 4(d) and 4(e)), the expression of NR4A3 (Figure 4(b)) and MMP13 proteins (Figures 4(i) and 4(j)), and the secretion of inflammatory factors IL-6 and TNF-*α* (Figures 4(f) and 4(g)), while NR4A3 overexpression reversed the alleviating effect of GSK3B overexpression on IL-1*β*-induced chondrocyte functions, suggesting that hypermethylation of NR4A3 promoter could improve the degeneration of chondrocytes induced by IL-1*β*.

### 3.5. NR4A3 Interference Alleviates IL-1*β*-Induced Degeneration of Mouse Chondrocytes by Inhibiting the JAK2/STAT3 Pathway

**Figure 5:**
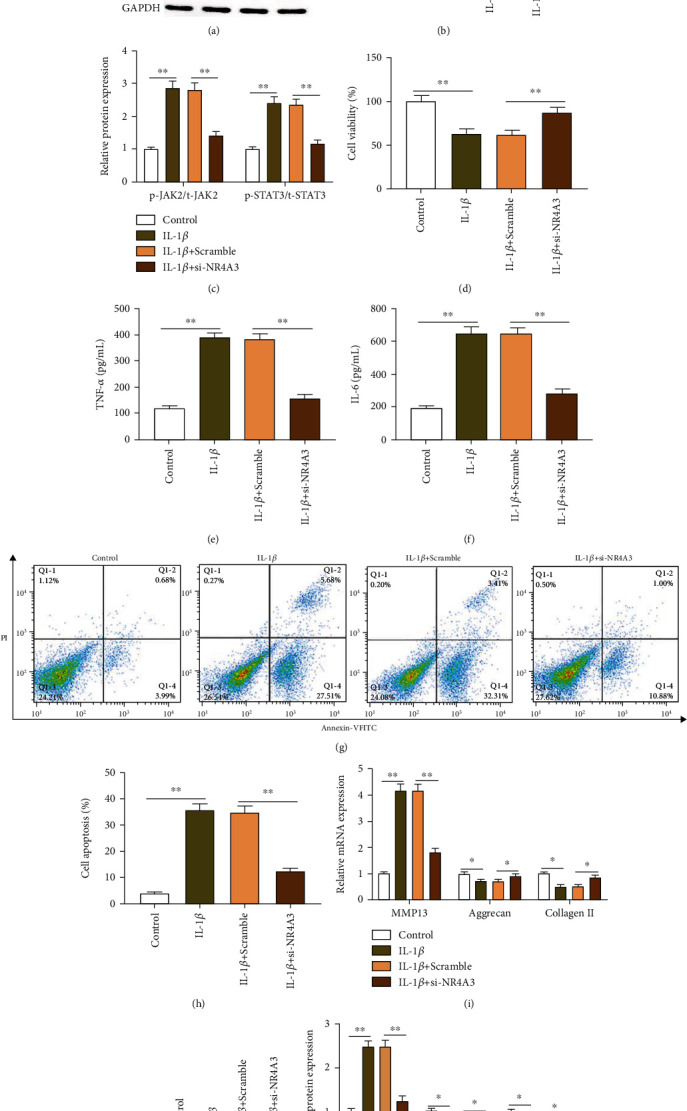
NR4A3 interference alleviates IL-1*β*-induced degeneration of mouse chondrocytes by inhibiting the JAK2/STAT3 pathway. IL-1*β*-induced chondrocytes were transfected with 200 nM si-NR4A3 or corresponding negative control for 48 h. (a–c, j, k) Western blotting was used to detect the expression of NR4A3, phosphorylated JAK2 (p-JAK2), JAK2, p-STAT3, STAT3, MMP13, aggrecan, and type II collagen proteins. (d) CCK-8 assay was used to detect cell viability. (e, f) Flow cytometry was used to detect apoptosis. (g, h) ELISA kits were used to detect the secretion of inflammatory factors TNF-*α* and IL-6. (i) RT-qPCR was used to detect the expression of MMP13, aggrecan, and type II collagen mRNA. Data is presented as the means ± SD for six independent experiments and analyzed by Student's *t*-test or ANOVA test. ^∗^*P* < 0.05 and ^∗∗^*P* < 0.01.

Previous studies demonstrated that inhibiting the activation of the JAK2/STAT3 pathway could alleviate the degradation of osteoarthritis chondrocytes [15]. In the current study, in order to explore the downstream mechanism of NR4A3, we performed NR4A3 interference experiments in mouse chondrocytes and then tested the cell functions. Western blotting results displayed that compared with the control group, IL-1*β* induction dramatically promoted the phosphorylation of JAK2 and STAT3 proteins in chondrocytes (Figures 5(a) and 5(c)), and NR4A3 interference dramatically decreased the expression of NR4A3 (Figures 5(a) and 5(b)), phosphorylated JAK2 and STAT3 proteins (Figures 5(a) and 5(c)), as well as the expression of aggrecan and collagen II proteins (Figures 5(j) and 5(k)), and increased the expression of MMP13 protein (Figures 5(j) and 5(k)). Simultaneously, NR4A3 interference significantly alleviated the decrease in chondrocyte viability induced by IL-1*β* (Figure 5(d)) and the increase of apoptosis (Figures 5(e) and 5(f)) and the secretion of inflammatory factors TNF-*α* and IL-6 (Figures 5(g) and 5(h)). RT-qPCR results displayed that NR4A3 interference significantly reduced the expression of aggrecan and collagen II mRNA (Figure 5(i)) and increased the expression of MMP13 mRNA (Figure 5(i)). The results above suggested that NR4A3 interference could alleviate the degradation of IL-1*β*-induced chondrocytes by inhibiting the phosphorylation of JAK2 and STAT3 proteins.

### 3.6. GSK3B Promotes NR4A3 Promoter Methylation by Recruiting DNMT1 to Improve Cartilage Mineralization in PTOA Mice

**Figure 6:**
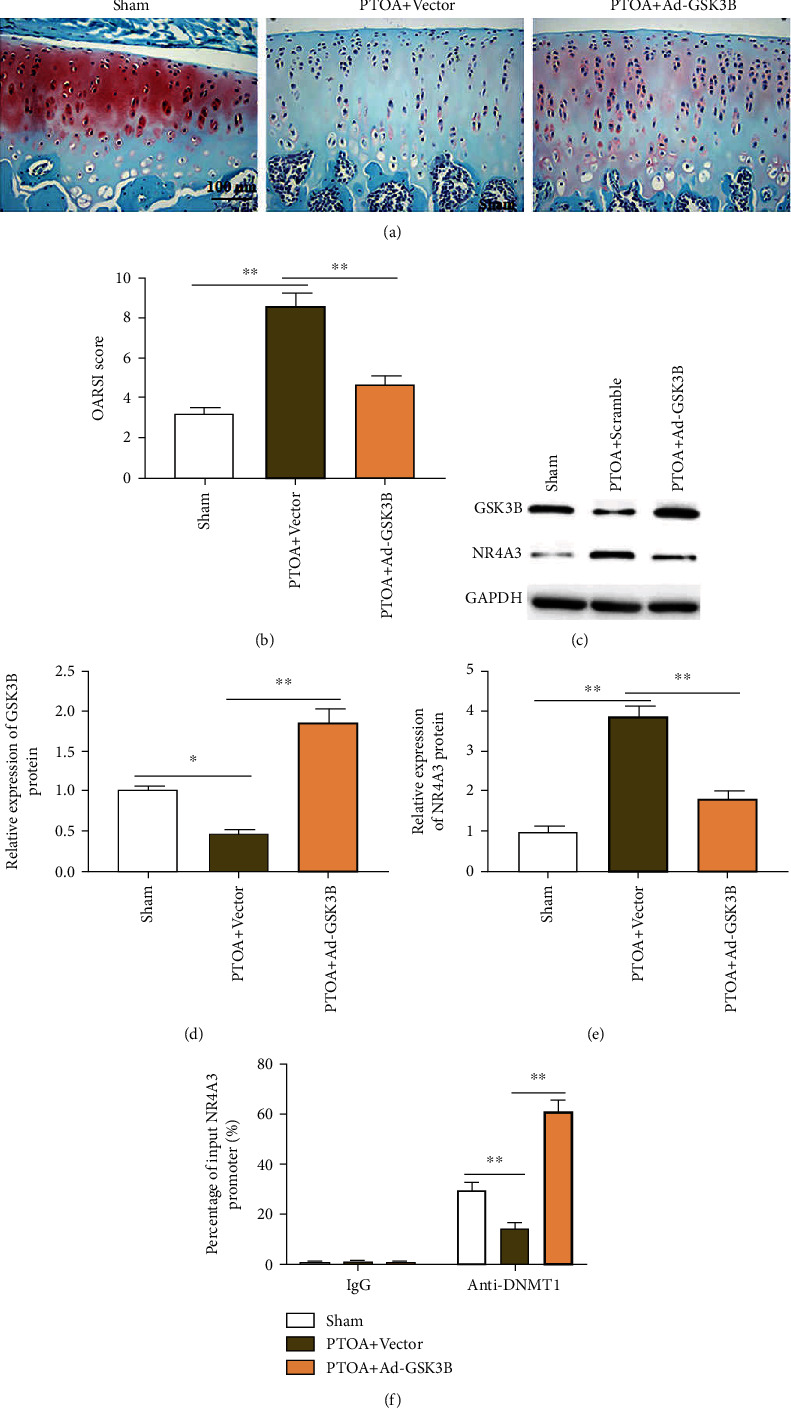
GSK3B promotes NR4A3 promoter methylation by recruiting DNMT1 *in vivo*. C57BL/6 mice were randomly divided into 5 groups: sham, posttraumatic oleanolic acid (PTOA)+vector, PTOA+adeno-related virus GSK3B overexpression vector (Ad-GSK3B), PTOA+scramble, and PTOA+sh-NR4A3, 10 mice per group. Destabilization of medial meniscus (DMM) surgery was performed on mice to generate a posttraumatic OA model. Immediately after the surgery, the joint was injected with 100 *μ*L of the following adeno-related virus vectors (empty vector, Ad-GSK3B, scrambled shRNA, and sh-NR4A3), respectively, and the virus titer was 2 × 10^8^ TU/mL. (a) Safranin O/fast green staining was used to assess cartilage damage in the sham, PTOA+vector, and PTOA+Ad-GSK3B groups. (b) Mouse OARSI score. (c–e) Western blotting was used to detect the expression of GSK3B and NR4A3 proteins in mouse knee joint tissues. (f) The change of DNMT1 bind to the promoter of NR4A3 by chromatin immunoprecipitation (Ch-IP) method. Data is presented as the means ± SD and analyzed by Student's *t*-test or ANOVA test. *n* = 10, ^∗^*P* < 0.05 and ^∗∗^*P* < 0.01.

The adeno-associated virus GSK3B overexpression vector was injected into the joint cavity of mice to study the role of GSK3B in the pathogenesis of PTOA. Massive loss of chondrocytes in the mouse joints, thickening of calcified cartilage, and loss of proteoglycan were observed in the PTOA+vector group mice when compared to the sham group using safranin O/fast green staining (Figure 6(a)). Simultaneously, the OARSI score of cartilage damage was significantly increased (Figure 6(b)), the expression level of GSK3B protein in joint tissues was significantly reduced (Figures 6(c) and 6(d)), and the expression level of NR4A3 protein was significantly increased (Figures 6(c) and 6(e)). Compared with the PTOA+vector group, the cartilage mineralization of PTOA+Ad-GSK3B group mice was significantly ameliorated (Figure 5(a)), the OARSI score and the expression level of NR4A3 protein in joint tissues were significantly reduced (Figures 6(b), 6(c), and 6(e)), and the expression level of GSK3B protein was significantly increased (Figures 6(c) and 6(d)). To investigate the effect of GSK3B on the expression of NR4A3 *in vivo*, we collected mouse knee joint tissues and performed Ch-IP assay and found that PTOA significantly decreased the level of DNMT1 bound to the promoter of NR4A3 compared with the sham group. In the PTOA+Ad-GSK3B group, overexpression of GSK3B significantly increased the level of DNMT1 bound to the promoter of NR4A3 compared with the PTOA+vector group (Figure 6(f)).

### 3.7. NR4A3 Interference Alleviates Cartilage Degradation in PTOA Mice

**Figure 7:**
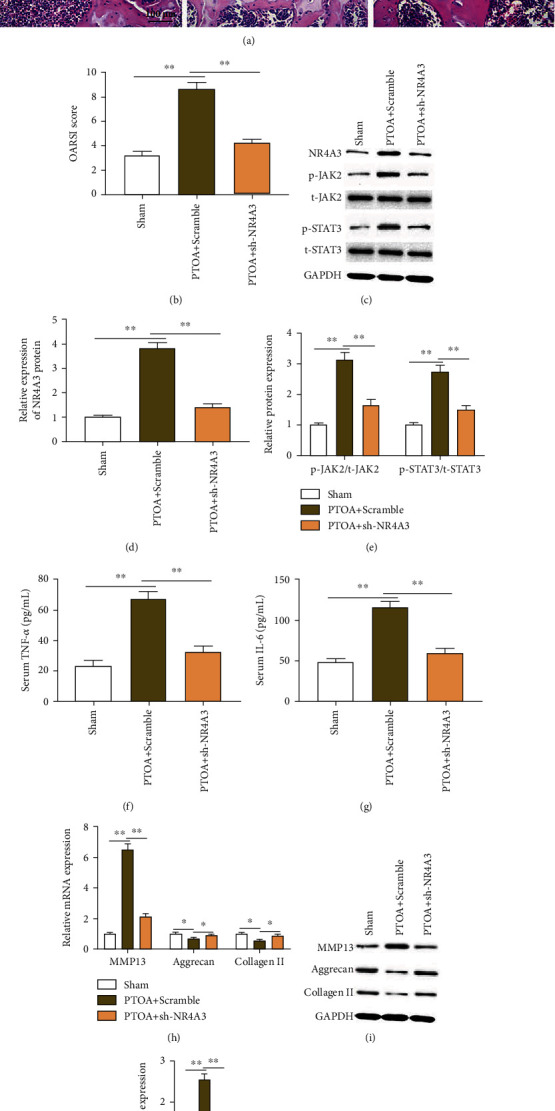
NR4A3 interference alleviates cartilage degradation in PTOA mice. (a) H&E and safranin O/fast green staining was used to assess cartilage damage in sham, PTOA+scramble, and PTOA+sh-NR4A3 groups. (b) Mouse OARSI score. (c–e, i, j) Western blotting was used to detect the expression of NR4A3, p-JAK2, JAK2, p-STAT3, STAT3, MMP13, aggrecan, and type II collagen proteins in mouse knee joint tissues. (f, g) ELISA kits were used to detect the secretion of inflammatory factors TNF-*α* and IL-6. (h) RT-qPCR was used to detect the expression of MMP13, aggrecan, and type II collagen mRNA. Data is presented as the means ± SD and analyzed by Student's *t*-test or ANOVA test. *n* = 10, ^∗^*P* < 0.05 and ^∗∗^*P* < 0.01.

To investigate the effect of NR4A3 *in vivo*, PTOA mice were injected with adenovirus-mediated NR4A3 shRNA to interfere with NR4A3. The results of safranin O/fast green and H&E staining showed that compared to the sham group, the thickening of calcified cartilage, loss of proteoglycan, destruction of the surface integrity of articular cartilage, massive loss of cartilage cells, and disordered arrangement were observed in PTOA+scramble group mice (Figure 7(a)). Meanwhile, compared to the sham group, the mouse joint tissue OARSI score (Figure 7(b)), the expression of NR4A3 protein (Figures 7(c) and 7(d)) and phosphorylated JAK2 and STAT3 proteins (Figures 7(c) and 7(e)), the expression of MMP13 mRNA and protein (Figures 7(h)–7(j)), and the level of serum inflammatory factors TNF-*α* and IL-6 were significantly increased in PTOA+scramble group mice (Figures 7(f) and 7(g)), and the expression of aggrecan and collagen II mRNA and proteins was significantly reduced (Figures 7(h)–7(j)). Compared with the PTOA+scramble group, the cartilage mineralization of PTOA+sh-NR4A3 group mice was significantly ameliorated (Figure 5(a)), and the OARSI score (Figure 7(b)), the expression of NR4A3 protein (Figures 7(c) and 7(d)), the expression of phosphorylated JAK2 and STAT3 proteins (Figures 7(c) and 7(e)), the expression of MMP13 mRNA and protein (Figures 7(h)–7(j)), and the level of serum inflammatory factors TNF-*α* and IL-6 in joint tissues were significantly reduced (Figures 7(f) and 7(g)), and the expression of aggrecan and collagen II mRNA and proteins was significantly increased (Figures 7(h)–7(j)). The above experimental results suggested that GSK3B inhibits the expression of NR4A3 and activation of the JAK2/STAT3 pathway by recruiting DNMT1 to the NR4A3 promoter region *in vivo*, thereby alleviating cartilage degradation in PTOA mice.

## 4. Discussion

OA is a disease characterized by articular cartilage degradation, subchondral bone changes, osteophyte formation, and synovitis. It is one of the major health burdens in the industrialized world. About half of the patients who have experienced joint trauma such as tibial plateau fractures, meniscus tears, or ruptured anterior cruciate ligaments (ACL) will develop PTOA [18]. Compared with spontaneous OA, PTOA is especially common among young people and could have long-term adverse effects on the quality of life [19]. GSK3B inactivation is considered to be a dangerous event in OA, which could lead to chronic damage of cartilage cells in the joint by aggravating inflammation [20]. GSK3B regulates the typical Wnt signaling pathway, which is essential for the maintenance of chondrocyte phenotype. In osteoarthritis, the decrease of GSK3B activity leads to the accumulation of *β*-catenin protein in the cytoplasm. However, after nuclear translocation of *β*-catenin protein, it could be used as a cofactor of TCF/LEF transcription factor to induce the expression of Wnt target genes. Inhibition of GSK3B in chondrocytes *in vitro* could lead to the loss of cartilage marker expression and chondrocyte apoptosis [21]. In the current study, we found that the expression of GSK3B protein in cartilage tissue of patients with PTOA was downregulated compared to the normal control group. And the expression of GSK3B protein was downregulated in IL-1*β*-induced mouse primary chondrocytes. Simultaneously, GSK3B overexpression could promote IL-1*β*-induced chondrocyte viability and the expression of cartilage extracellular matrix markers aggrecan and type II collagen and inhibit cell apoptosis, the secretion of inflammatory factors TNF-*α* and IL-1*β*, and the expression of MMP13.

Recent methylome studies revealed the differential DNA methylation characteristics of patients with OA. It shows that this kind of epigenetic regulation of DNA structure changes may be an important factor in the development and progress of OA [22, 23]. The dynamic DNA methylation process consists of DNA methyltransferase (DNMT) enzymes and demethylases. Three common types of DNMT (DNMT1, 3A, and 3B) catalyze the addition of methyl (CH_3_) to the cytosine located at the 5′end of guanine (CpG site) to form methylated cytosine and silence the target gene [24]. A matched case-control study showed that DNMT1, DNMT3A, and DNMT3B gene polymorphisms are significantly associated with radioactive primary knee osteoarthritis. Among them, the rs2228611 and rs2228612 genotypes of DNMT1 are associated with reducing the risk of primary knee OA [25]. GSK3B silencing could prevent histone H3 phosphorylation and reduce DNMT1 expression, thereby inhibiting the proliferation of breast cancer cells induced by high glucose, and the expression of GSK3B and DNMT1 proteins are in the same direction in breast cancer cells [26]. In the current study, we verified the interaction between GSK3B and DNMT1 using Co-IP experiments.

Our current research explored the downstream mechanism of GSK3B regulation in PTOA. An existing study found that NR4A3 may be one of the many DNA methylation genes involved in the pathogenesis of migraine [27]. Moreover, a study established an acute skeletal muscle contraction model by stimulating differentiated C2C12 cells with electrical pulses. This study used bisulfite sequencing and found that in response to electrical pulse stimulation, a region of the NR4A3 promoter was rapidly demethylated at 60 minutes and remethylated at 120 minutes [28]. Only one study explored the upregulation of NR4A3 in cartilage tissues of OA patients and IL-1*β*-induced rat chondrocytes. And the high expression of NR4A3 enhanced the expression of chondrocyte matrix degradation genes at the protein and mRNA levels [12], while the role and underlying mechanism of NR4A3 in PTOA are still unclear. In this study, we found that in chondrocytes, GSK3B overexpression could motivate the methylation of NR4A3 and suppress the expression of NR4A3 by recruiting DNMT1 to the NR4A3 promoter region, and GSK3B interference had the opposite effect. Moreover, we performed NR4A3 interference in IL-1*β*-induced chondrocytes, and then, the cell functions were detected. To our surprise, NR4A3 silencing could activate the cell viability and inhibit IL-1*β*-induced chondrocyte apoptosis and extracellular matrix degradation.

Many proinflammatory cytokines, especially IL-1*β*, IL-6, and TNF-*α*, are prominently elevated in OA patients. These proinflammatory factors are believed to increase chondrocyte apoptosis and extracellular matrix degradation by activating the Janus kinase/signal transducers and activators of transcription (JAK/STAT) signaling pathway [29]. A study identified NR4A3 as the target gene of STAT3 in gastric cancer. And NR4A3 could activate STAT3 to inhibit the progression of gastric cancer [30]. Our current study found that NR4A3 interference could reduce IL-1*β*-induced chondrocyte apoptosis and extracellular matrix degradation by inhibiting the activation of the JAK2/STAT3 pathway. *In vivo*, overexpression of GSK3B could inhibit the expression of NR4A3 protein by recruiting DNMT1 to the NR4A3 promoter region, thereby ameliorating cartilage mineralization in PTOA mice, which has almost the same effect as NR4A3 interference.

Taken together, this study explores the role and internal connection of GSK3B and NR4A3 in the pathogenesis of PTOA. We newly discovered that GSK3B recruits DNMT1 to the NR4A3 promoter region and inhibits the activation of the NR4A3-mediated JAK2/STAT3 signaling pathway, thereby alleviating PTOA. This provides a new perspective for us to understand the pathological mechanism of PTOA. Unfortunately, this study has certain limitations, that is, the histological analysis of mice is not comprehensive and in-depth. In the future, we would use microcomputed tomography, tartrate-resistant acid phosphatase staining, and immunohistochemistry to analyze the pathological features and molecular levels of articular cartilage in PTOA model mice.

## Data Availability

The datasets used during the present study are available from the corresponding author on reasonable request.

## Abbreviations

PTOA: Posttraumatic osteoarthritis
GSK3B: Glycogen synthase kinase 3*β*
NR4A3: Nuclear receptor subfamily 4 group A member 3
DMEM: Dulbecco's modified Eagle medium
PBS: Phosphate-buffered saline
FBS: Fetal bovine serum
DMM: Destabilization of medial meniscus
OARSI: Osteoarthritis Research Society International
ECM: Extracellular matrix
IL-6: Interleukin-6
TNF-*α*: Tumor necrosis factor *α*
Co-IP: Coimmunoprecipitation
JAK/STAT: Janus kinase/signal transducers and activators of transcription.
